# AglQ Is a Novel Component of the *Haloferax volcanii* N-Glycosylation Pathway

**DOI:** 10.1371/journal.pone.0081782

**Published:** 2013-11-13

**Authors:** Adi Arbiv, Sophie Yurist-Doutsch, Ziqiang Guan, Jerry Eichler

**Affiliations:** 1 Department of Life Sciences, Ben Gurion University, Beersheva, Israel; 2 Department of Biochemistry, Duke University Medical Center, Durham, North Carolina, United States of America; University of Alberta, Canada

## Abstract

N-glycosylation is a post-translational modification performed by members of all three domains of life. Studies on the halophile *Haloferax volcanii* have offered insight into the archaeal version of this universal protein-processing event. In the present study, AglQ was identified as a novel component of the pathway responsible for the assembly and addition of a pentasaccharide to select Asn residues of *Hfx. volcanii* glycoproteins, such as the S-layer glycoprotein. In cells deleted of *aglQ*, both dolichol phosphate, the lipid carrier used in *Hfx. volcanii* N-glycosylation, and modified S-layer glycoprotein Asn residues only presented the first three pentasaccharide subunits, pointing to a role for AglQ in either preparing the third sugar for attachment of the fourth pentasaccharide subunit or processing the fourth sugar prior to its addition to the lipid-linked trisaccharide. To better define the precise role of AglQ, shown to be a soluble protein, bioinformatics tools were recruited to identify sequence or structural homologs of known function. Site-directed mutagenesis experiments guided by these predictions identified residues important for AglQ function. The results obtained point to AglQ acting as an isomerase in *Hfx. volcanii* N-glycosylation.

## Introduction

Although the ability of Archaea to perform protein N-glycosylation in Archaea was first reported in 1976 [1], it was only in the last decade that efforts focused on delineating the pathways involved in the archaeal version of this universal post-translational modification, thanks to the development of appropriate molecular tools. Today, many of the details of the N-glycosylation process are known for several Archaea, including the halophile, *Haloferax volcanii*. 

In *Hfx. volcanii*, a series of *agl* (archaeal *gl*ycosylation) genes encode proteins involved in the assembly and attachment of a pentasaccharide to select Asn residues of the reporter glycoprotein, the surface (S)-layer glycoprotein. Acting at the cytoplasmic face of the plasma membrane, AglJ, AglG, AglI, AglE sequentially add the first four pentasaccharide residues (i.e. a hexose, two hexuronic acids and the methyl ester of a hexuronic acid) onto a common dolichol phosphate (DolP) carrier, while AglD adds the final pentasaccharide residue, mannose, to a distinct DolP [2-8]. In addition, N-glycosylation roles have been assigned to AglF, a glucose-1-phosphate uridyltransferase [9], AglM, a UDP-glucose dehydrogenase [9] and AglP, a methyltransferase [8]. Once assembled, the glycan-charged lipids are translocated across the membrane by an unknown mechanism involving AglR [10]. Then, in a reaction requiring the oligosaccharyltransferase, AglB [2], the tetrasaccharide and its precursors are delivered to select Asn residues of the S-layer glycoprotein. Finally, the terminal pentasaccharide residue, mannose, is transferred from its DolP carrier to the protein-bound tetrasaccharide by the actions of AglS [11]. 

Apart from AglD, all of the currently known components of the *Hfx. volcanii* N-glycosylation pathway are encoded by sequences sequestered to a common gene island [12]. However, in addition to containing genes of known function, the *agl* gene cluster also includes several sequences whose contribution to N-glycosylation remains only poorly defined. For example, AglQ (HVO_1523; GenBank accession number CAW30728.1) has been implicated in N-glycosylation based on the co-transcription of its encoding gene with the neighboring *aglP* sequence, encoding a known N-glycosylation pathway component [8,12]. Still, although expressed [12], no function had been assigned to AglQ. In the present study, a combination of gene deletion, mass spectrometry, bioinformatics and biochemical approaches show AglQ to be involved in the appearance of the methyl ester of hexuronic acid found at position four of the pentasaccharide N-linked to the S-layer glycoprotein, possibly acting as an isomerase.

## Materials and Methods

### Strains and growth conditions

The *Hfx. volcanii* parent strain WR536 (H53) and the isogenic strain deleted of *aglQ* were grown in medium containing 3.4 M NaCl, 0.15 M MgSO_4_•7H_2_0, 1 mM MnCl_2_, 4 mM KCl, 3 mM CaCl_2_, 0.3 % (w/v) yeast extract, 0.5 % (w/v) tryptone, 50 mM Tris-HCl, pH 7.2, at 40°C [13]. 

### Deletion of aglQ

Deletion of *Hfx. volcanii aglQ* was achieved using a previously described approach in with the *aglQ* sequence is replaced by the tryptophan synthase-encoding *trpA* sequence in a tryptophan auxotrophic strain [1,4]. To amplify approximately 500 bp-long regions flanking the coding sequence of *aglQ*, the aglQ-5’upfor (gggctcgagCGACTCGTTTACTAATATGC; genomic sequence in capital letters) and aglQ-5’uprev (cccaagcttTGTTCCTCCGATCTTAGG) primers, directed against the upstream flanking region, and the aglQ-3’downfor (gggggatccACAACAAAAAAGACGAACTATTG) and aglQ-3’downrev (ccctctagaGAGGGCTCGAATGAGATATCC) primers, directed against the downstream flanking region, were employed. *Xho*I and *Hind*III sites were introduced using the aglQ-5'upfor and aglQ-5'uprev primers, respectively, while *Bam*HI and *Xba*I sites were introduced using the aglQ-3’downfor and aglQ-3’downrev primers, respectively.

To confirm deletion of *aglQ* at the DNA level, PCR amplification was performed using forwards primers directed against either an internal region of *algQ* (aglQ-for; ATGACCTCTCTTTCTGACATTCTTGC) or *trpA* (cccgaattcTTATGTGCGTTCCGGATGCG) together with a reverse primer against a region downstream of *aglQ* (aglQ-5’downrev), respectively yielding primer pairs a and b, or using primers aglQ-for and aglQ-rev (TTAGTCAAGATATATCTCGTAGTC), designed to amplify a section of the *aglQ* coding region (primer pair c). Reverse-transcription (RT)-PCR was performed as described previously [1], using primer pair c to test for *algQ* transcription, so as to confirm *aglQ* deletion at the RNA level.

### Mass spectrometry

Normal phase liquid chromatography-electrospray ionization mass spectrometry (LC-ESI MS) analysis of a total *Hfx. volcanii* lipid extract and of the S-layer glycoprotein was performed as described previously [13,14]. 

### Proteolytic digestion of the *Hfx*. *volcanii* S-layer

S-layer resistance to proteolysis of cells of the *Hfx. volcanii* parent strain and of *Hfx. volcanii* Δ*aglQ* cells (1 ml) was assessed as described previously [7].

### Sub-cellular fractionation

*Hfx. volcanii* cells (1 ml) expressing GFP-tagged AglQ were broken by sonication (2 s on and 1 s off for 90 s, 25% output, Misonix XL2020 ultrasonicator). Unbroken cells were pelleted in a microfuge (9,000 x g, 10 min, 4°C) and the resulting supernatant was centrifuged in an ultracentrifuge (Sorvall M120; 240,000 x g, 12 min, 4 °C). While the resulting supernatant was directly precipitated in 15% (w/v) tri-chloroacetic acid, the pelleted membrane fraction was resuspended in 200 µl of distilled water and then precipitated in 15% (w/v) tri-chloroacetic acid. Proteins were electrotransferred from SDS-PAGE gels to nitrocellulose membranes (0.45 µm, Schleicher & Schuell, Dassel, Germany) and incubated with anti-GFP (1:1,000; Roche) or anti-SRP54 antibodies (1:10,000) [16]. Binding of these primary antibodies was detected using horseradish peroxidase (HRP)-conjugated goat anti-mouse antibodies (1:2,500; KPL, Gaithersburg, MD) or goat anti-rabbit antibodies (1:4,000; BioRad), respectively, and ECL Western blotting detection reagent (GE Healthcare). The distribution of the S-layer glycoprotein was determined by Coomassie-staining of the cytosolic and membrane protein pools in SDS-PAGE gels.

### Site-directed mutagenesis

Mutated versions of *aglQ* were generated by site-directed mutagenesis using the Quikchange (Stratagene) protocol, performed according to the manufacturer’s instructions. Oligonucleotide primers used to introduce the various mutations are listed in Table S1. To generate constructs encoding the AglQ mutants fused to GFP, DNA sequences corresponding to the various mutants were PCR amplified using forward and reverse primers (forward primer: 5’-gggtctagaATGACCTCTCTTTCTGACATT-3’; reverse primer: 5’-cccagatctTTAGTCAAGATATATCTCG-3’) designed to introduce *Xba*I and *BglI*I restriction sites at the 5’- and 3’-ends of the fragments, respectively. The amplified fragments were digested with *Xba*I and *BglI*I, purified by electrophoresis in 1 % agarose gels and ligated into plasmid pJAM1020 [17], pre-digested with the appropriate restriction enzymes to yield plasmids encoding the various AglQ mutants, with the promoter normally found in the plasmid being replaced by the 200 bp region lying upstream to each open reading frame being considered. The introduction of mutations into *aglQ* was confirmed by sequencing, performed both before and following vector introduction into *Hfx. volcanii* Δ*aglQ* cells. To assess the levels of expression of the different GFP-tagged AglQ mutants, immunoblotting was performed using anti-GFP antibodies, HRP-conjugated goat anti-mouse antibodies and ECL Western blotting detection reagent.

## Results

### N-glycosylation is perturbed in *Hfx*. *volcanii* cells deleted of aglQ

Given the co-transcription of *aglQ* with *aglP* [12], the latter encoding a confirmed component of the *Hfx. volcanii* N-glycosylation pathway [8], efforts were directed at defining the precise contribution of AglQ to this post-translational modification. Accordingly, *Hfx. volcanii* cells were deleted of the encoding gene and the resulting effect on N-glycosylation of the S-layer glycoprotein, a reporter of N-glycosylation, was considered by mass spectrometry.

The *aglQ* gene was deleted from the *Hfx. volcanii* genome and replaced by the *Hfx. volcanii* tryptophan synthase-encoding *trpA* sequence via the ‘pop-in/pop-out’ approach developed by Allers et al. [18]. Replacement of *aglQ* by *trpA* was verified at the DNA level by PCR, using genomic DNA from the parent or the deletion strain as template, together with a forward primer directed at a region within *aglQ* and a reverse primer directed at a downstream region or using the same reverse primer together with a forward primer directed at a region within *trpA*. While the parent strain contains the *aglQ* sequence, the gene was not detected in the deletion strain, having been replaced by the *trpA* sequence (not shown).

**Figure 1:**
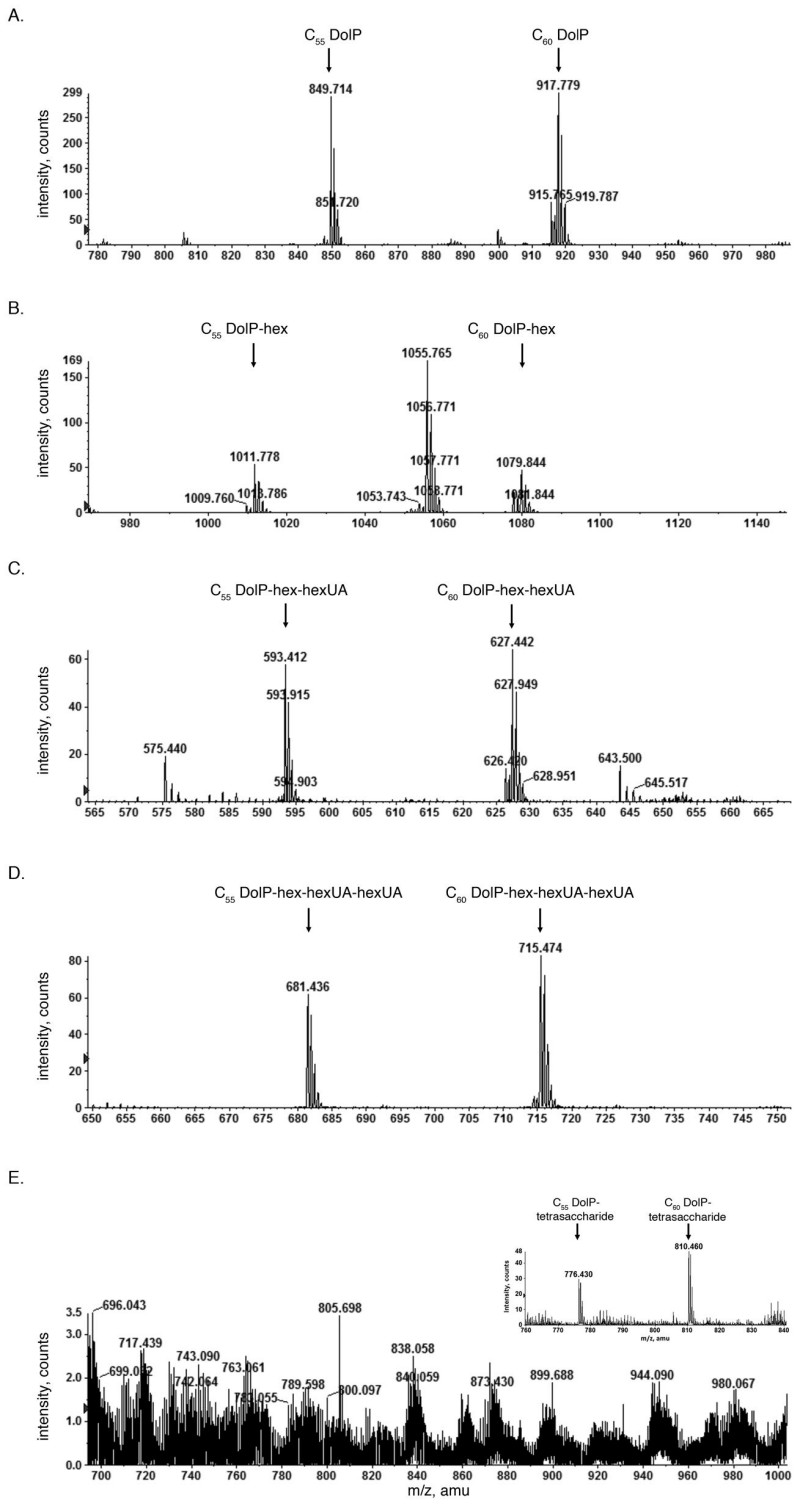
DolP glycosylation in compromised in Δ*aglQ* cells. Normal phase LC-ESI MS analysis of a total *Hfx*. *volcanii* lipid extract revealed [M-H]^-^ ions corresponding to (A) C_55_ and C_60_ DolP, as well as the same lipids modified by (B) hexose (C_55_ and C_60_ DolP-hex). [M-2H]^2-^ ions corresponding to the same lipids modified by (C) hexose and a hexuronic acid (C_55_ and C_60_ DolP-hex-hexUA) and (D) hexose and two hexuronic acids (C_55_ and C_60_ DolP-hex-hexUA-hexUA) were also detected, as indicated. E. Δ*aglQ* cells do not contain tetrasaccharide-charged DolP, unlike cells of the parent strain, where [M-2H]^2-^ ions corresponding to DolP modified by hexose, two hexuronic acids and a methyl ester of hexuronic acid are seen (C_55_ and C_60_ DolP-tetrasaccharide) are readily detected (inset).

In *Hfx. volcanii*, DolP serves as the lipid carrier upon which a pentasaccharide N-linked to the S-layer glycoprotein is assembled. Such assembly involves the sequential addition of the first four pentasaccharide residues to a common DolP; the final pentassacharide residue, mannose, is added to a distinct DolP [6]. To assess the effect of *aglQ* deletion at the glycan-charged DolP level, a total *Hfx. volcanii* lipid extract was subjected to normal phase LC-ESI MS, as reported previously [6]. Such analysis revealed prominent ion peaks of *m/z* 849.714 (this and all reported values are for the monoisotopic ion peaks, unless otherwise stated) and *m/z* 917.779, corresponding to the [M-H]^-^ ions of C_55_ and C_60_ DolP, each containing two saturated isoprene units, respectively (Figure 1A). In addition, C_55_ and C_60_ dolichol phosphate species modified by a hexose (peaks at *m/z* 1011.778 and 1079.844, respectively) were observed (Figure 1B). The same profile also revealed a major peak at the *m/z* 1055.765, corresponding to a previously described *Hfx. volcanii* sulfoglycolipid, S-GL-1 [19]. [M-2H]^2-^ ions of the C_55_ and C_60_ dolichol phosphate species modified by a hexose and a hexuronic acid (peaks at *m/z* 593.412 and 627.442, respectively) (Figure 1C) and by a hexose and two hexuronic acids (peaks at *m/z* 681.436 and 715.474, respectively) (Figure 1D) were also observed. However, no C_55_ and C_60_ dolichol phosphate species modified by a hexose, two hexuronic acids and a methyl ester of hexuronic acid, i.e. the first four residues of the pentasaccharide N-linked to the S-layer glycoprotein, were detected in the lipid extract prepared from cells deleted of *aglQ* (Figure 1E). At the same time, in control experiments in which the same extract prepared from cells of the parent strain was examined by LC-ESI MS, [M-2H]^2-^ ions at *m/z* 766.430 and 810.460, corresponding to tetrasaccharide-charged C_55_ and C_60_ dolichol phosphate, respectively, were readily detected (Figure 1E, inset). As such, it appears that cells lacking AglQ are only able to assembly trisaccharide-charged DolP.

**Figure 2:**
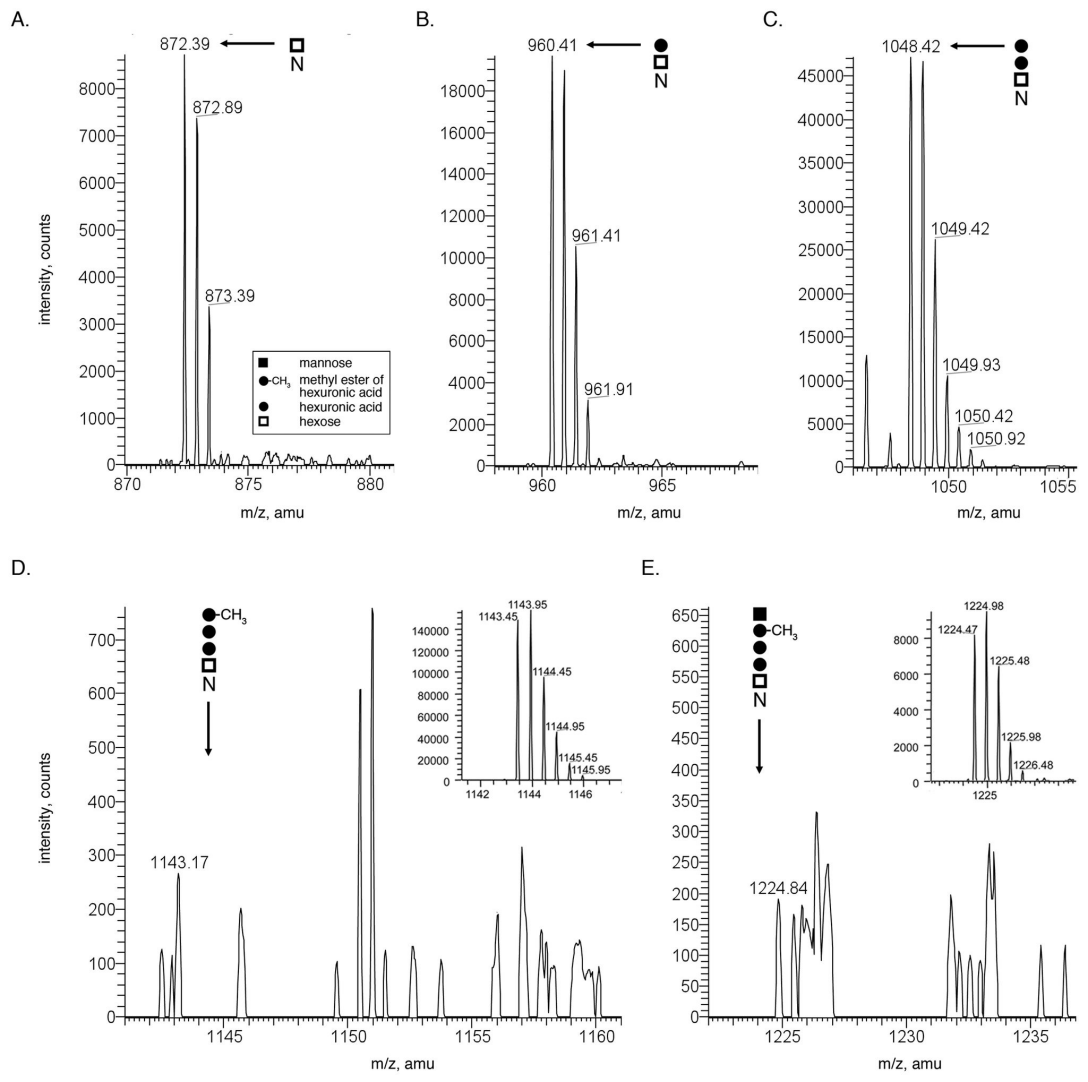
S-layer glycoprotein N-glycosylation in compromised in Δ*aglQ* cells. Following trypsin treatment, the S-layer glycoprotein of Δ*aglQ* cells was examined by normal phase LC-ESI MS. Shown are profiles obtained for the ^1^ERGNLDADSESFNK^14^ glycopeptide. Arrows indicate the positions of [M+2H]^2+^ ions corresponding to the peptide modified by (A) the first, (B) the first two and (C) the first three sugar residues of the pentasaccharide normally N-linked to this position. No [M+2H]^2+^ ion peaks corresponding to the same peptide modified by the first four pentasaccharide residues (D) nor the complete pentasaccharide (E) were detected in the *aglQ* deletion strain, despite such species being readily detected in the same sample obtained from parent strain cells (insets of D and E, respectively). The identity of each pentasaccharide subunit is provided in the inset in (A).

Next, N-glycosylation of the S-layer glycoprotein was assessed in cells of the *aglQ* deletion strain. Normal phase LC-ESI MS analysis of a deletion strain S-layer glycoprotein-derived tryptic peptide, ^1^ERGNLDADSESFNK^14^, previously shown to be N-glycosylated at the Asn-13 position [20,2], revealed [M+2H]^2+^ ions corresponding to the peptide modified by the first (*m/z* 872.32; Figure 2A), the first two (*m/z* 960.41; Figure 2B) and the first three (*m/z* 1048.42; Figure 2C) sugar residues of the pentasaccharide normally N-linked to this position. No [M+2H]^2+^ ion peaks corresponding to the same peptide modified by the first four pentasaccharide residues (Figure 2D) nor the complete pentasaccharide (Figure 2E) were detected in the *aglQ* deletion strain, despite such species being readily detected in the same sample obtained from parent strain cells ([M+2H]^2+^ ions at *m/z* 1143.45 and *m/z* 1224.47 presented in the insets to Figures 2D and 2E, respectively). Thus, reminiscent of what was seen in terms of DolP glycosylation, *aglQ* deletion also results in N-glycosylation of S-layer glycoprotein by a trisaccharide, rather than the pentasaccharide that decorates this reporter glycoprotein in parent strain cells.

**Figure 3:**
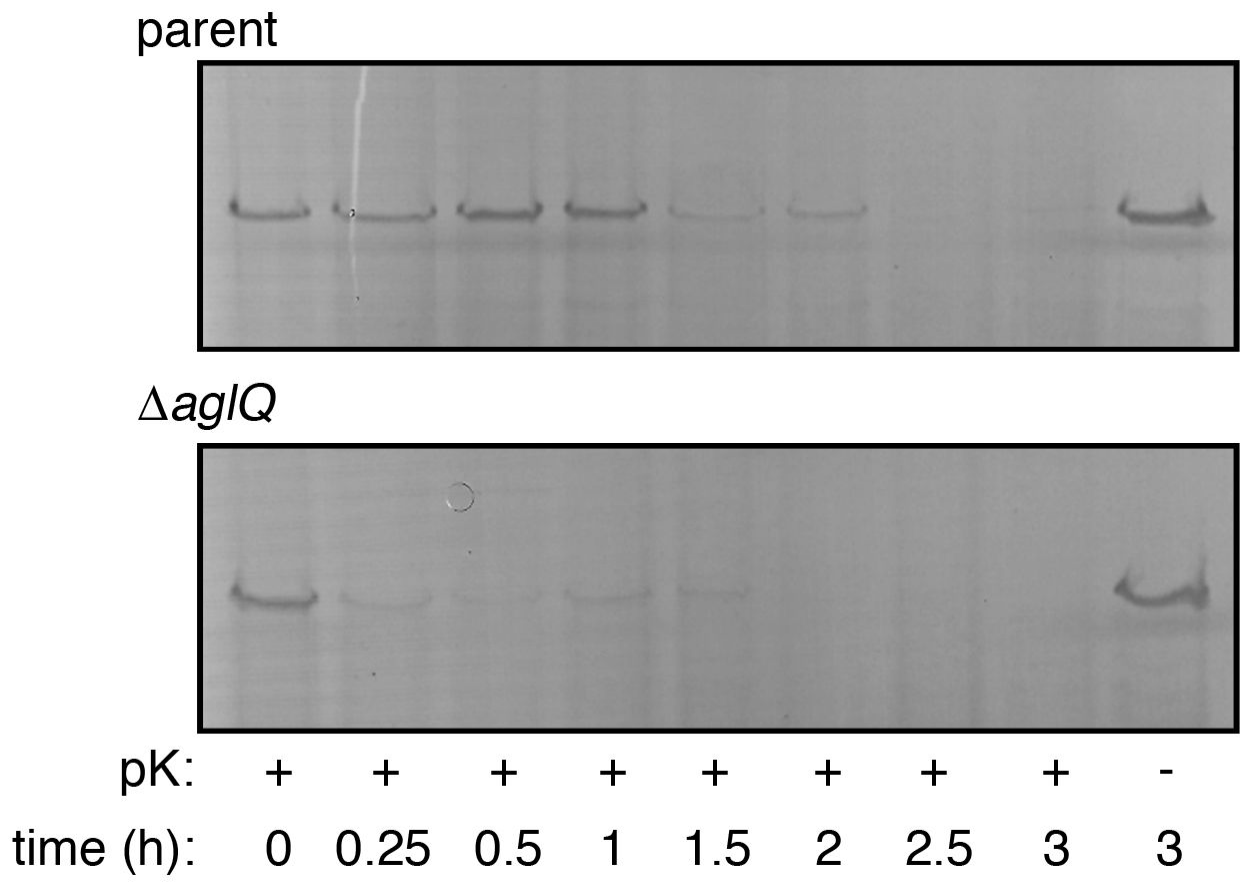
S-layer integrity in compromised in Δ*aglQ* cells. Parent strain (top panel) and Δ*aglQ* cells (lower panel) were challenged with 1 mg/ml proteinase K at 42°C. Aliquots were removed immediately prior to incubation with proteinase K and at 15-30 min intervals following addition of the protease for up to 3 h and examined by 7.5% SDS-PAGE and Coomassie staining. The S-layer glycoprotein band from each gel is presented.

In *Hfx. volcanii*, the S-layer glycoprotein is the sole component of the proteinaceous S-layer surrounding the cell [20]. Hence, the effect of deleting *aglQ* on S-layer stability was addressed. Previous studies have shown that cells lacking AglF, AglG, AglI, AglJ and AglM present a S-layer this is more susceptible to proteolytic degradation than is the same protein array in cells of the parent strain [5,7,15]. Just as with these other mutants lacking components of the *Hfx. volcanii* N-glycosylation pathway, cells lacking AglQ also possess an S-layer that was more rapidly degraded when challenged with added proteinase K than was the S-layer of parent strain cells. While a 1.5 h incubation with proteinase K was needed to digest the bulk of the S-layer glycoprotein pool of parent strain cells, a similar degree to proteolysis in the AglQ-lacking cells was seen after an incubation as short as 15 min (Figure 3). Such enhanced sensitivity to proteolysis is likely due to defects in N-glycosylation resulting from the absence of AglQ.

### AglQ is a soluble protein

**Figure 4:**
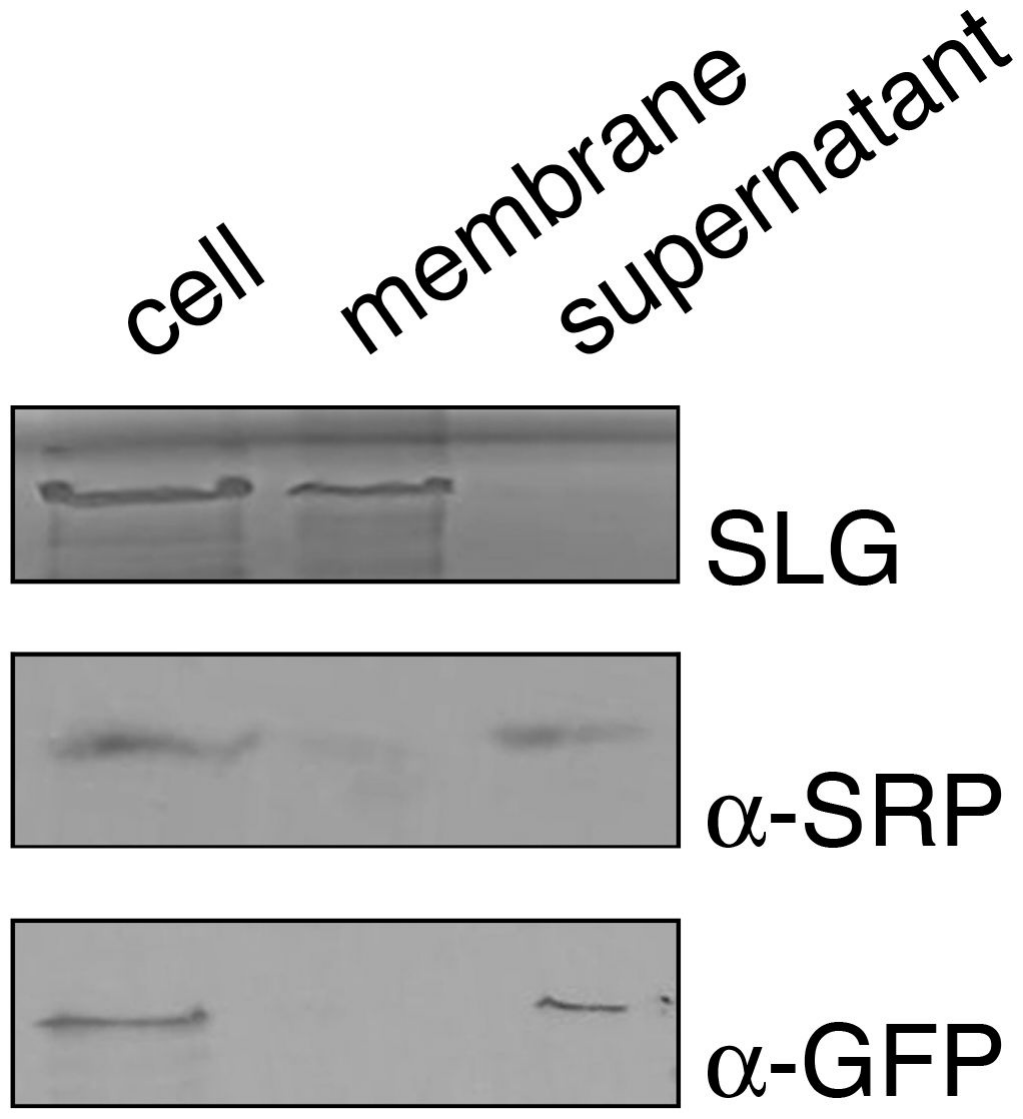
AglQ is a soluble protein. *Hfx*. *volcanii* cells transformed to express GFP-AglQ were separated into membrane and cytosolic (supernatant) fractions and probed with anti-GFP (α-GFP) or anti-SRP54 (α-SRP54) antibodies, as was a total protein extract (cell). Alternatively, the position of the S-layer glycoprotein in the same fractions was identified by Coomassie staining. The proteins migrate with the following molecular masses in SDS-PAGE: S-layer glycoprotein, 180 kDa [20], SRP-54, 51 kDa [16], GFP-AglQ, 44 kDa.

To begin defining the function of AglQ in the *Hfx. volcanii* N-glycosylation pathway, efforts next focused on defining the sub-cellular localization of the protein. Accordingly, the HMMTOP (http://www.enzim.hu/hmmtop/), SOSUI (http://bp.nuap.nagoya-u.ac.jp/sosui/), TMHMM (http://www.cbs.dtu.dk/services/TMHMM-2.0/) TopPred (http://bioweb.pasteur.fr/seqanal/interfaces/toppred.html) and TMpred (http://www.ch.embnet.org/software/TMPRED_form.html) topology prediction servers were consulted. In each case, AglQ was designated as a soluble protein. To directly test this prediction, *Hfx. volcanii* cells were transformed to express a GFP-tagged version of AglQ. The transformed cells were disrupted by sonication and the resulting lysate was separated into soluble and membrane fractions by ultracentrifugation. When each fraction was then probed for the presence of GFP-AglQ in an immunoblot protocol using anti-GFP antibodies, the chimera was exclusively localized to the soluble fraction (Figure 4). In control experiments, the S-layer glycoprotein, a marker of the plasma membrane [20], and SRP54, a marker of the cytoplasm [16], were localized to the membrane and soluble fractions, respectively, confirming the efficacy of the sub-cellular fractionation performed here.

### Bioinformatics approaches identify residues possibly important for AglQ function

To gain further insight into the biological role of AglQ, a BLAST homology search was conducted. Twenty-one proteins were identified in several haloarchaeal or bacterial species as homologs with E-values less than 3e-5. At the time of this search (August, 2013), these sequences are all annotated as hypothetical proteins. In a PSI-BLAST search conducted using AglQ as query (five iterations; December, 2012 and repeated August, 2013), the vast majority of sequences recognized as homologs with E-values less the 1e-5 are listed as glucuronyl hydrolases or the more general glycosyl hydrolase family 88 members, alginate lyases or hypothetical proteins. As a glycosyl hydrolase family 88 member, glucuronyl hydrolase catalyzes the hydrolysis of oligosaccharides with unsaturated glucuronyl residues at the non-reducing terminal, while other glycosyl hydrolase family 88 members contribute to the degradation of oligosaccharides with an unsaturated uronic acid at the non-reducing terminal [13]. Alginate lyases catalyze the breakdown of alginate, a polymer of the sugars α-L-guluronate and its C-5 epimer, β-D-mannuronate [21]. Glycosyl hydrolase family 88 members and alginate lyases (PF05426 and PF07470, respectively) were also included in the list of six-best hits when the AglQ sequence was used to scan the Pfam database (http://pfam.sanger.ac.uk/). Such analysis, however, listed a N-acylglucosamine 2-epimerase family (PF07221) member, namely the enzyme that catalyzes the interconversion of N-acyl-D-glucosamine and N-acyl-D-mannosamine [22], as the top hit. 

Given the limited insight obtained from these primary sequence-based servers, more sophisticated bioinformatics tools were employed, when structure homology servers were consulted. As listed in Table 1, the top five structural homologs of AglQ identified (Dec., 2012) by Phyre2 (Protein Homology/AnalogY Recognition Engine; http://www.sbg.bio.ic.ac.uk/phyre2/html/page.cgi?id=index) or SCOP (Structural Classification of Proteins database; http://scop.mrc-lmb.cam.ac.uk/scop/) are all assigned various saccharide processing-related roles. Structure-based multiple alignments were then generated for the three highest scoring structural homologs identified, using the UCSF-Chimera program (https://www.rbvi.ucsf.edu/chimera/index.html). The first alignment was based on the model of AglQ creating using a N-acyl-D-glucosamine 2-epimerase structure (pdb 1fp3; [23]), the top ranked structural homolog identified by Phyre2 and the third best structural homolog ranked by SCOP, as template. The second was based on the model of AglQ created using the structure of an unsaturated glucuronyl hydrolase (pdb 2d5j; [24]), the top ranked structural homolog identified by SCOP and the fourth best structural homolog ranked by Phyre2, as template. Finally, the third multiple sequence alignment obtained was based on the AglQ model generated using the structure of YihS (pdb 2afa), corresponding to the second ranked structural homolog identified by SCOP and the fifth best structural homolog ranked by Phyre2, as template. The three alignments are presented in Figures S1, S2, S3. The various alignments were next scanned for identical or highly conserved residues, as these are assumed to be important for the catalytic activity of the different proteins, possibly including AglQ. In this manner, the AglQ residues His-34, Glu-37, Thr-38, Phe-50, Lys-52, Glu-55, Asp-58, Glu-59, Arg-61, Ala-66, His-81, Lys-93, Trp-104, Arg-114, Asn-118 and Asp-187 were selected for further consideration as being significant for the N-glycosylation-related function of the protein.

**Table 1:** Predicted structural homologues of AglQ.

**Phyre2**		**SCOP**	
**Protein**	**Structure (pdb)**	**Protein**	**Structure (pdb)**
*Sus scrofa* N-acyl-D-glucosamine 2-epimerase	1fp3	*Bacillus* sp. GL1 unsaturated glucuronyl hydrolase	2d5j
*Streptococcus agalactiae* unsaturated glucuronyl hydrolase	2zzr	*Salmonella typhimurium* N-acyl-glucosamine isomerase (YihS)	2afa
*Anabaena* sp. CH1 N-acetyl-D-glucosamine 2-epimerase	2gz6	*Sus scrofa* N-acyl-D-glucosamine 2-epimerase	1fp3
*Bacillus* sp. GL1 unsaturated glucuronyl hydrolase	2d5j	*Bacillus subtilis* lyase (YteR)	1nc5
*Salmonella typhimurium* N-acyl-glucosamine isomerase (YihS)	2afa	*Cellvibrio japonicas* polysaccharide lyase	1gxm

### Thr-38 and Lys-52 are important for AglQ function

To test the importance of those AglQ residues suspected of contributing to the activity of the protein on the basis of structure-based homology analysis, site-directed mutagenesis was performed. In such efforts, plasmids encoding GFP-tagged versions of the AglQ mutated at the positions listed above were introduced into *aglQ* deletion strain cells. The ability of each introduced mutant protein to restore AglQ activity was then tested by addressing the N-glycosylation profile of the S-layer glycoprotein from the transformed cells by LC-ESI MS. Mutations yielding a version of AglQ unable to restore the missing activity of the deletion strain thus reflect the mutated position as being important for AglQ function. The choice of mutation for a given residue was based on either the size or the charge of the original residue at that position, or by success in generating a particular mutant. 

**Figure 5:**
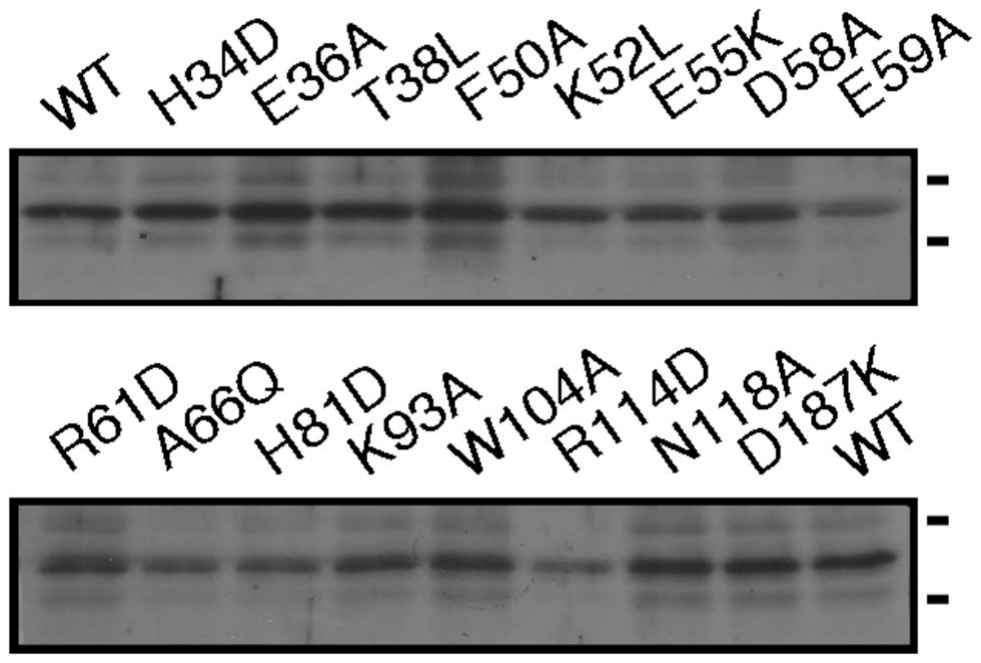
Expression of AglQ mutants. The levels of the different versions of AglQ fused to GFP generated following site-directed mutagenesis and expression in Δ*aglQ* cells is shown. The protein content of equivalent amounts of *Hfx*. *volcanii* Δ*aglQ* cells expressing the various AglQ mutants were separated by SDS-PAGE subjected to immunoblot using anti-GFP and appropriate secondary HRP-conjugated antibodies. The positions of 55 and 40 kDa molecular weight markers are depicted on the right of each panel.

LC-ESI MS analysis of a S-layer glycoprotein-derived Asn-13-containing tryptic fragment revealed that no loss of AglQ activity was observed when the GFP-tagged H34D, E37A, F50A, E55K, D58A, E59A, R61D, A66Q, H81D, W104A, R114D, N118A and D187K AglQ mutants were expressed in *aglQ* deletion strain cells (not shown). Since these mutations did not prevent AglQ activity, these residues were deemed as being non-crucial for the catalytic actions of the protein. By contrast, the T38L and K52L mutants were unable to restore AglQ activity to the deletion strain since in Δ*aglQ*, with only the trisaccharide-modified Asn-13-containing peptide being detected in cells expressing these mutants. When K93A AglQ was expressed in the deletion strain, only tetrasaccharide-modified Asn-13 was observed. It would thus appear that Thr-38 and Lys-52 are central to AglQ-mediated catalysis, while Lys-93 may contribute to such activity. Finally, to confirm that the inability of these AglQ mutants to restore AglQ activity in the deletion strain was not due to their poor expression in the transformed Δ*aglQ* cells, immunoblot analysis using anti-GFP antibodies was performed. Such analysis confirmed that GFP-tagged T38L, K52L and K93A AglQ were expressed at levels comparable to the levels of other AglQ mutants, as well as of the wild type (Figure 5), all shown to restore the activity of the enzyme in the deletion strain. 

## Discussion

In *Hfx. volcanii*, the Agl pathway serves to assemble and attach a pentasaccharide to select Asn residues of target proteins, including the S-layer glycoprotein and archaellins (cf. [25]). Although most of the steps comprising this biosynthetic process have been delineated, the roles of several pathway components remain to be defined. In the present report, one of these uncharacterized Agl pathway components, AglQ, was considered. 

Relying on a variety of experimental approaches, AglQ was shown to be a soluble protein involved in attachment of the fourth subunit of the N-linked pentasaccharide, a methyl ester of a hexuronic acid, to the DolP carrier charged with the first three subunits of the glycan, likely by participating in the biosynthesis or processing of the third or fourth sugars of this glycan. Specifically, AglQ may prepare the third sugar of the DolP-linked trisaccharide for attachment of the fourth pentasaccharide sugar or may process this fourth sugar prior to its addition to the lipid-linked trisaccharide. Indeed, in the absence of AglQ, the final pentasaccharide subunit, mannose, is not transferred from its own DolP carrier to the N-linked glycan comprising these three pentasaccharide subunits already added to target Asn residues in a reaction normally performed by AglS, a DolP-mannose mannosyltransferase [11]. As a result, S-layer glycoproteins bearing only a N-linked trisaccharide (as well as the precursor di- and monosaccharides) are observed in cells deleted of *aglQ*. 

To date, several enzymes involved in the processing of the fourth pentasaccharide subunit have already been identified. AglE is the glycosyltransferase responsible for adding hexuronic acid to the DolP-linked trisaccharide corresponding the first three pentasaccharide subunits, while AglP is a SAM-dependent methyltransferase that adds a methyl group to this hexuronic acid after its linkage to the DolP-bound trisaccharide [4,8]. Finally, AglM, the only *Hfx. volcanii* nucleotide sugar dehydrogenase studied to date [9], is thought to convert UDP-hexose into the UDP-hexuronic acid that is subsequently processed by AglE and AglP. In the context of the Agl pathway, AglQ would, therefore, act after AglM yet before AglE. 

Despite the results obtained in this study, the precise role of AglQ remains unclear. A sequence homology-based search aimed at defining AglQ function identified a limited number of archaeal homologues, all encoded by halophilic species, as well as by a few bacterial species. In none of these organisms has a role been assigned to the AglQ homolog. Indeed, apart from *Halobacterium salinarum* [1], none of the other organisms encoding an AglQ homolog is known to perform N-glycosylation. In *Hbt. salinarum*, however, the gene encoding the AglQ homolog OE2533F is found in a region of the genome spanning OE2528R to OE2551F that includes homologues of *Hfx. volcanii aglB*, *aglE*, *aglF*, *aglG*, *aglI*, *aglJ*, *aglM*, *aglP*, *aglQ* and *aglR*, genes participating in the N-glycosylation pathway of this organism [9,12]. More sophisticated bioinformatics tools, including a search of the Pfam database and structure-based homology searches, revealed the similarity of AglQ to various epimerases or other isomerases, as well as glucuronyl hydrolases. Based on these predictions, site-directed mutagenesis approaches identified two AglQ residues (Thr-38 and Arg-93) found at common positions in the structures of these isomerases and in a model AglQ structure as being important for AglQ function. A third residue important for AglQ function, Lys-52, was identified based on the homology model of a glucuronyl hydrolase. The Y38L and K52L mutations led to an apparent loss of AglQ function since in cells expressing these mutants, the S-layer glycoprotein was only modified by the first three sugars of the pentasaccharide normally N-linked to this protein. However, in the case of the K93A mutation, a glycan comprising the first four pentasaccharide sugars was attached to the S-layer glycoprotein. This observation is consistent with K93A AglQ catalyzing the processing of the third sugar of the DolP-linked trisaccharide to be able to accept a different fourth sugar than is normally added at this position, and which cannot serve as a substrate for AglS, the enzyme responsible for adding mannose to the N-linked tetrasaccharide (11). This fourth sugar is, however, methylated by AglP (8). Alternatively, the mutant AglQ could fail to properly process the fourth sugar added to the DolP-linked trisaccharide, resulting in the same outcome. 

Further support for AglQ corresponding to an isomerase comes from recent studies showing that *Haloarcula marismortui* also N-glycosylates its S-layer glycoprotein with a pentasaccharide similar to that linked to glycoproteins in *Hfx. volcanii* [26]. *Har. marismortui*, however, does not encode an AglQ homolog. As such, the mass spectrometry approach used to reveal the similar compositions of the N-linked glycans decorating the S-layer glycoproteins of these two Dead Sea-derived haloarchaea would not be expected to detect isomerase/epimerase-mediated differences between the two pentasaccharides as catalyzed by AglQ in *Hfx. volcanii* but not in *Har. marismortui*. At the same time, the various bioinformatics approaches also reported the resemblance of AglQ to hydrolases and other enzymes able to release glucuronic acids from polysaccharides. It is, however, difficult to imagine how such hydrolytic activity would be relevant to the Agl pathway, and in particular, to the contribution of AglQ to this pathway. 

Should AglQ act as an epimerase or another isomer-generating enzyme, it could either modify the hexuronic acid that corresponds to the third subunit of the N-pentasaccharide so as to allow the addition of the hexuronic acid found at position four of the glycan (before the latter is methylated) or it could modify that hexuronic acid before it can be added to the DolP-bound trisaccharide. Ongoing efforts to define the precise composition of the N-linked pentasaccharide decorating glycoproteins in *Hfx. volcanii* will help discern between these two possibilities.

## Supporting Information

Figure S1 **Alignment based on the structure of AglQ generated using pdb 1fp3 as template.** A model of AglQ was created by the Chimera program using a N-acyl-D-glucosamine 2-epimerase structure (pdb 1fp3; Itoh et al., 2000) as template. A structure-based multiple alignment was generated using the Chimera program, with the positions of AglQ residues listed in the line listed as d1fp3a_.pdb. The vertically framed and orange shaded residues represent overlapping positions in the first structure used to generate the alignment, while the horizontally framed residues represent those missing from the coordinates section of the pdb file. The residues are color-coded according to the Clustal X coloring scheme (www.jalview.org/help/html/colourSchemes/clustal.html).
(DOCX)

Figure S2 **Alignment based on the structure of AglQ generated using pdb 2d5j as template.** A model of AglQ was created by the Chimera program using the structure of an unsaturated glucuronyl hydrolase (pdb 2d5j; Itoh et al., 2006) as template. A structure-based multiple alignment was generated using the Chimera program, with the positions of AglQ residues listed in the line listed as d2d5ja1.4.pdb. The vertically framed and orange shaded residues represent overlapping positions in the first structure used to generate the alignment, while the horizontally framed residues represent those missing from the coordinates section of the pdb file. The residues are color-coded according to the Clustal X coloring scheme (www.jalview.org/help/html/colourSchemes/clustal.html).
(DOCX)

Figure S3 **Alignment based on the structure of AglQ generated using pdb 2afa as template.** A model of AglQ was created by the Chimera program using the structure of YihS (pdb 2afa) as template. A structure-based multiple alignment was generated using the Chimera program, with the positions of AglQ residues listed in the line listed as d2afaa1.5.pdb. The vertically framed and orange shaded residues represent overlapping positions in the first structure used to generate the alignment, while the horizontally framed residues represent those missing from the coordinates section of the pdb file. The residues are color-coded according to the Clustal X coloring scheme (www.jalview.org/help/html/colourSchemes/clustal.html).
(DOC)

Table S1 **Primers used for site-directed mutagenesis.**
(DOC)
