# Supplementary figures and images for: AglQ Is a Novel Component of the Haloferax volcanii N-Glycosylation Pathway
Source: PLoS One. 2013 Nov 13;8(11):e81782. doi: 10.1371/journal.pone.0081782 (PMC3827465; doi:10.1371/journal.pone.0081782)

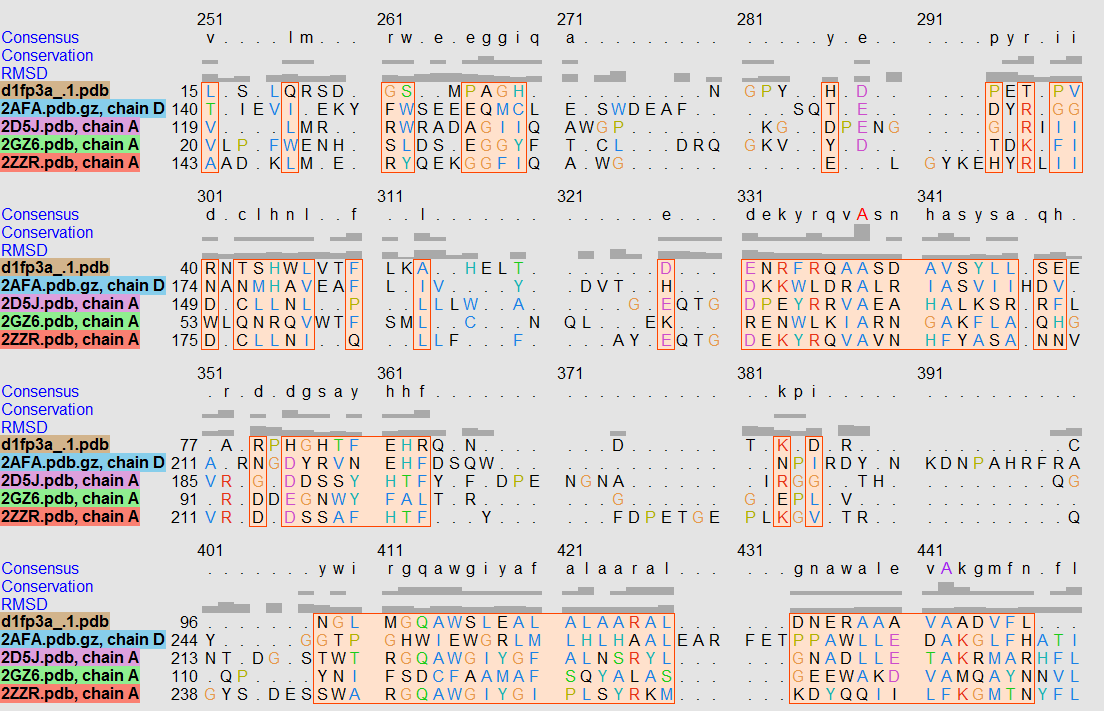

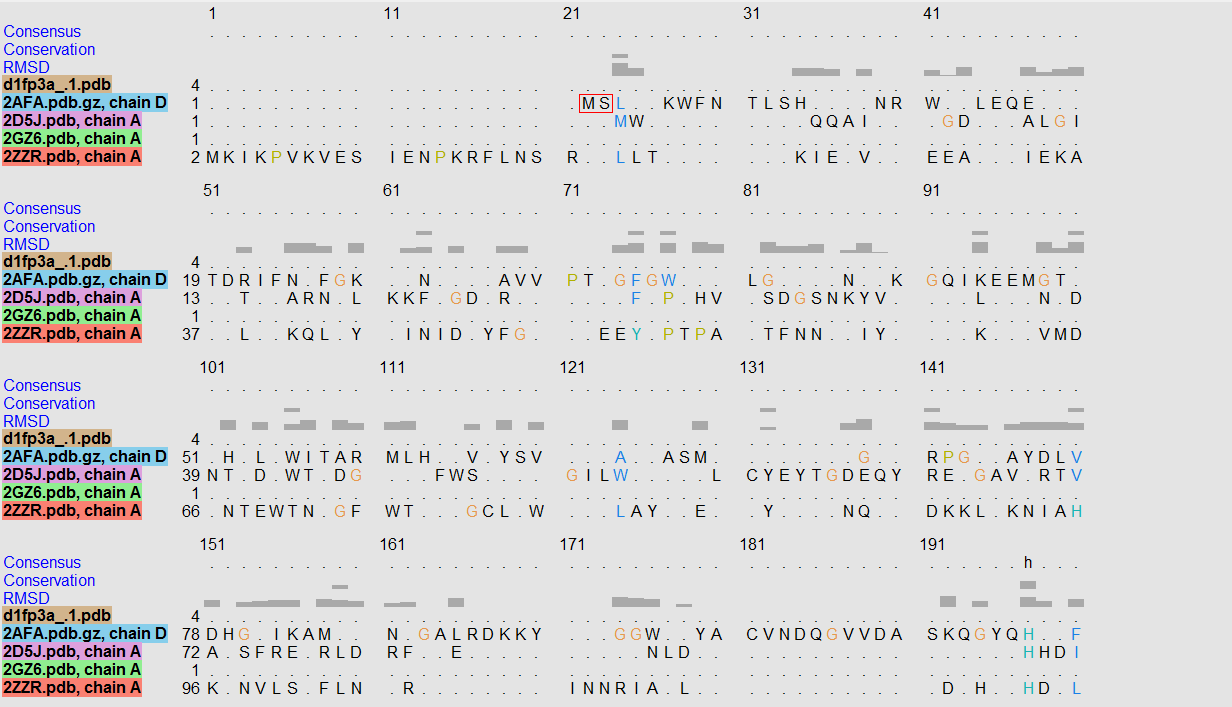
Fig S1


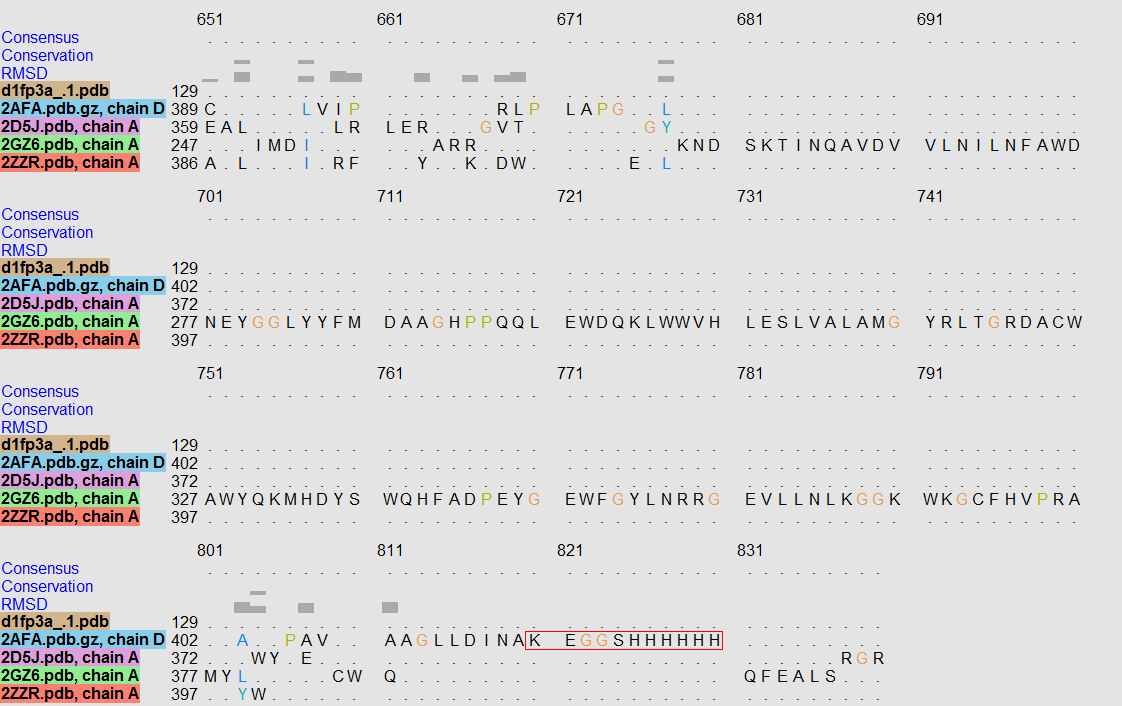
**
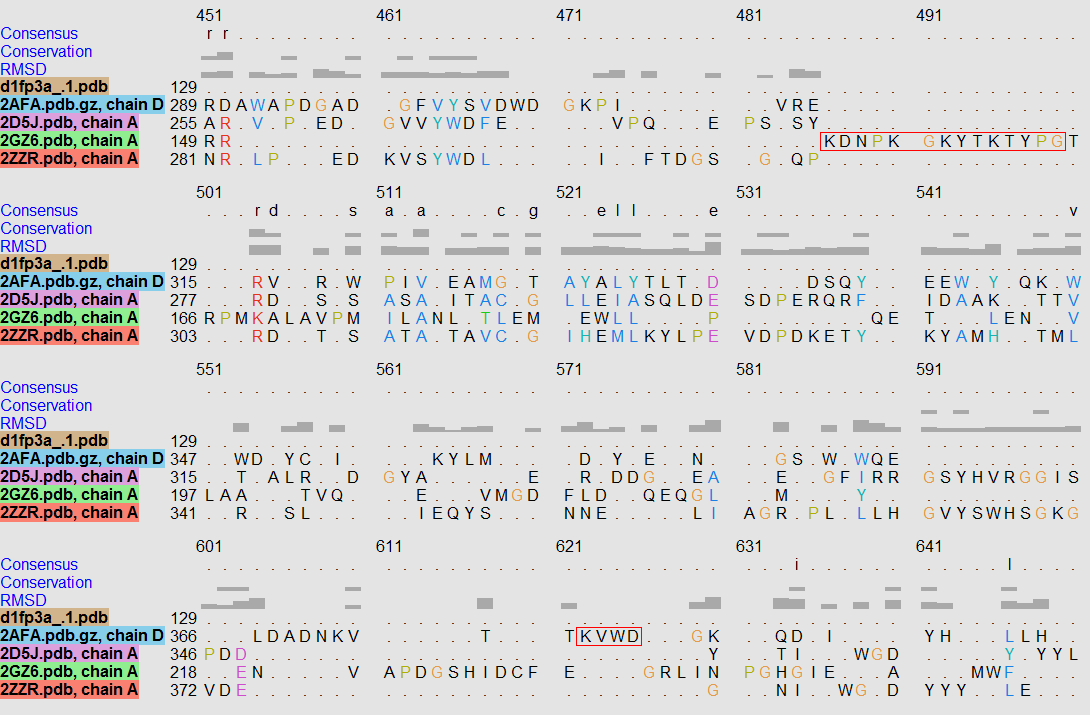
**

Supplement: Figure S1 — Alignment based on the structure of AglQ generated using pdb 1fp3 as template. A model of AglQ was created by the Chimera program using a N-acyl-D-glucosamine 2-epimerase structure (pdb 1fp3; Itoh et al., 2000) as template. A structure-based multiple alignment was generated using the Chimera program, with the positions of AglQ residues listed in the line listed as d1fp3a_.pdb. The vertically framed and orange shaded residues represent overlapping positions in the first structure used to generate the alignment, while the horizontally framed residues represent those missing from the coordinates section of the pdb file. The residues are color-coded according to the Clustal X coloring scheme (www.jalview.org/help/html/colourSchemes/clustal.html). (DOCX) [file pone.0081782.s001.docx]

Fig S2


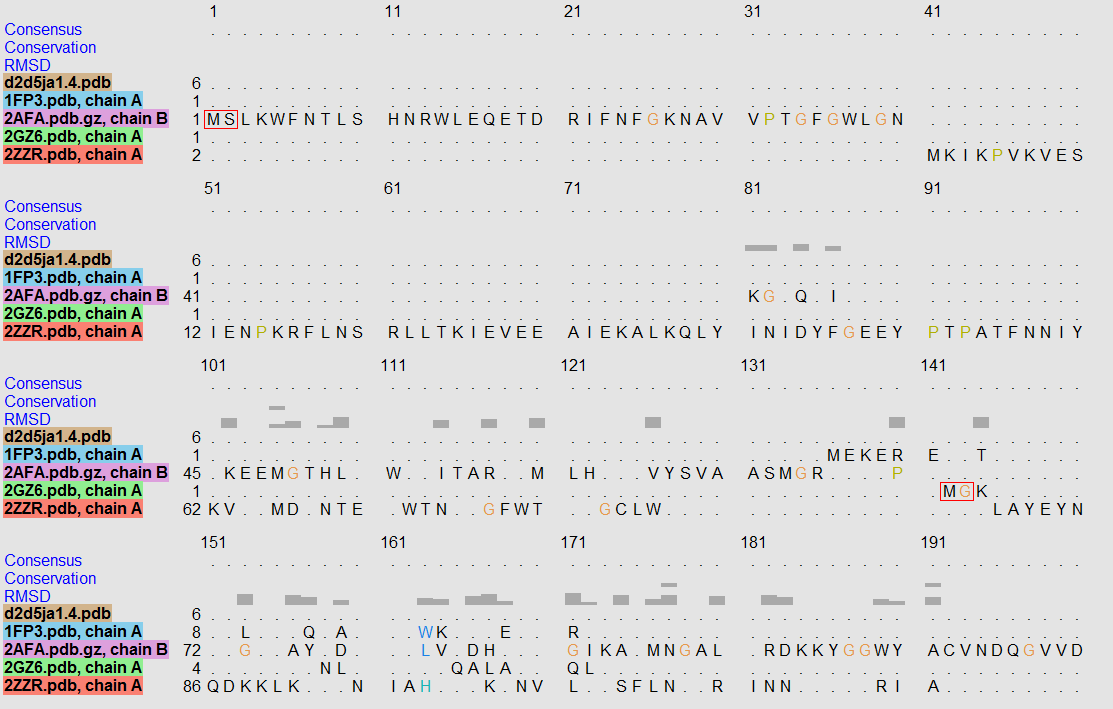

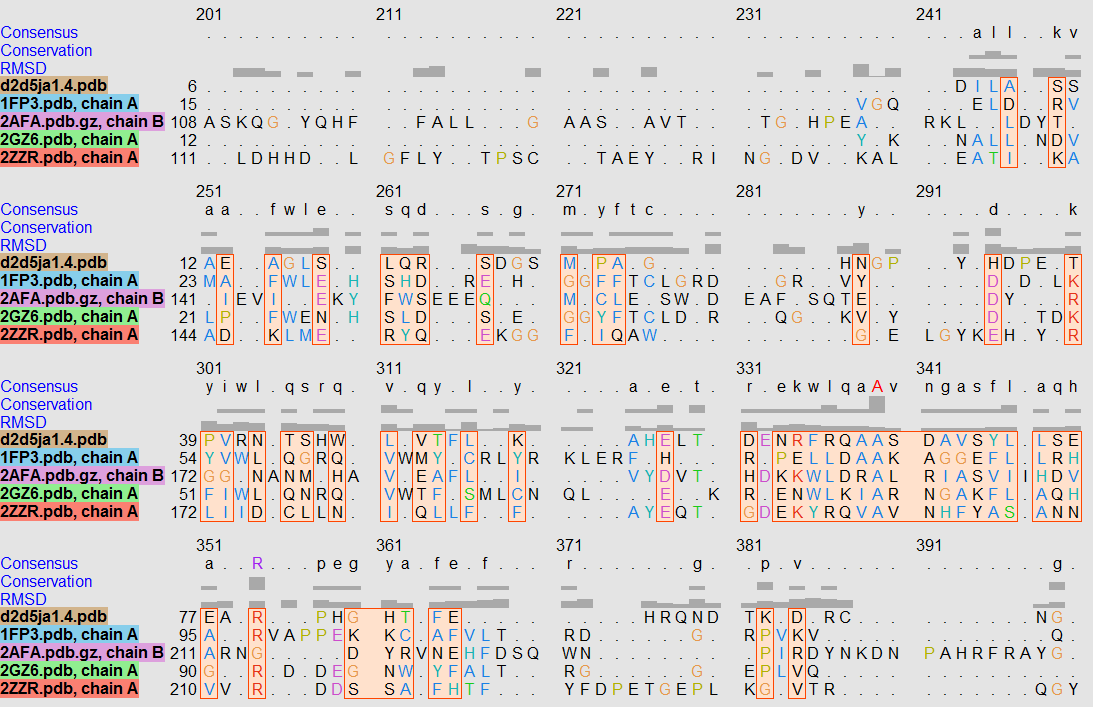


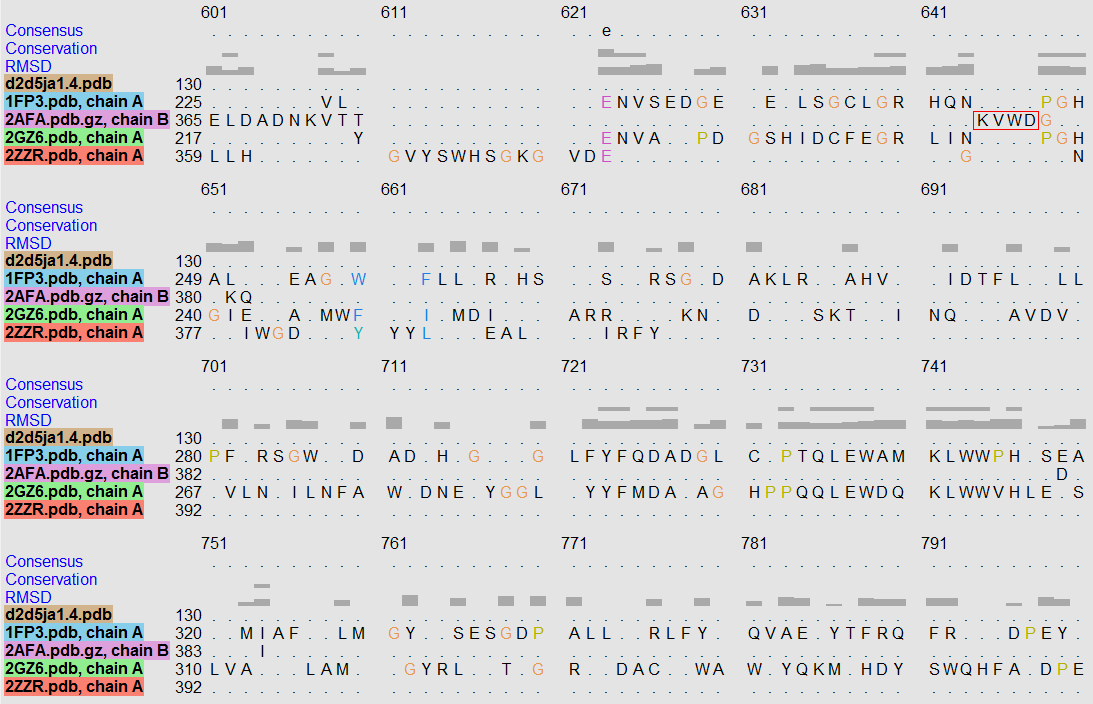

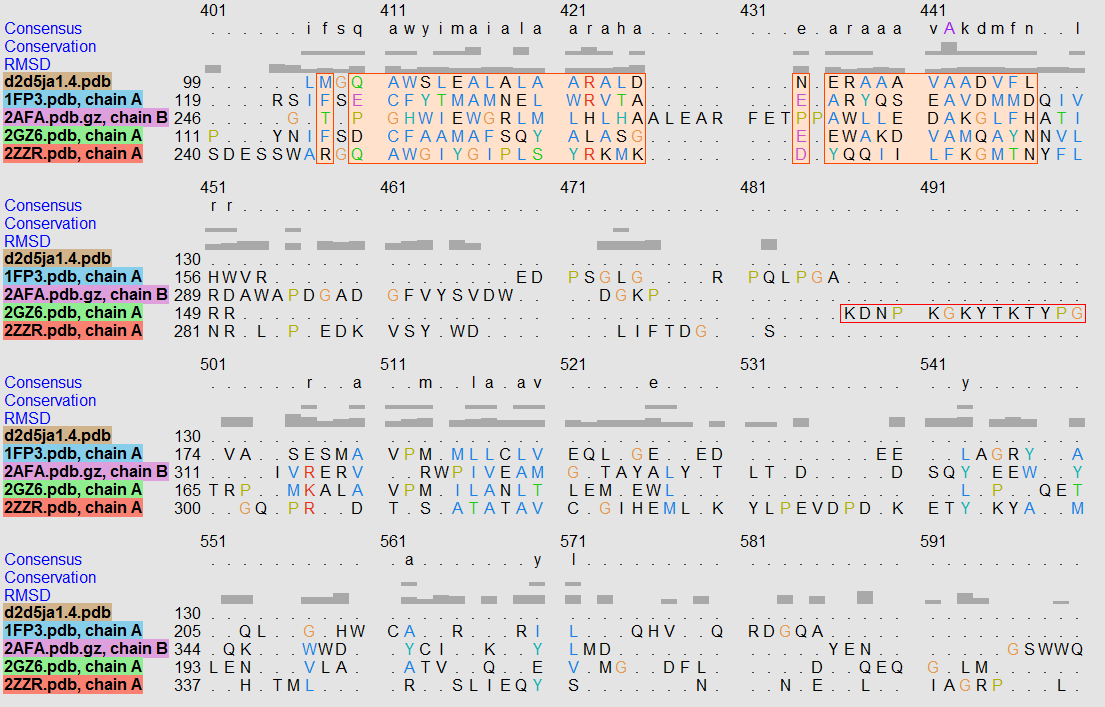


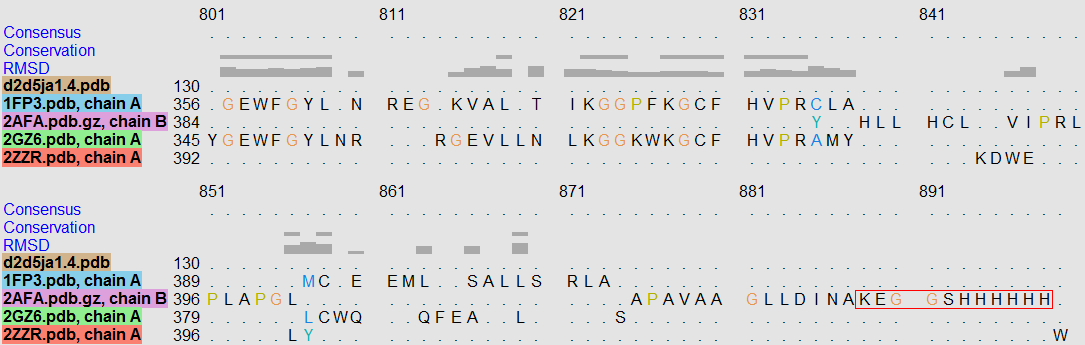

Supplement: Figure S2 — Alignment based on the structure of AglQ generated using pdb 2d5j as template. A model of AglQ was created by the Chimera program using the structure of an unsaturated glucuronyl hydrolase (pdb 2d5j; Itoh et al., 2006) as template. A structure-based multiple alignment was generated using the Chimera program, with the positions of AglQ residues listed in the line listed as d2d5ja1.4.pdb. The vertically framed and orange shaded residues represent overlapping positions in the first structure used to generate the alignment, while the horizontally framed residues represent those missing from the coordinates section of the pdb file. The residues are color-coded according to the Clustal X coloring scheme (www.jalview.org/help/html/colourSchemes/clustal.html). (DOCX) [file pone.0081782.s002.docx]

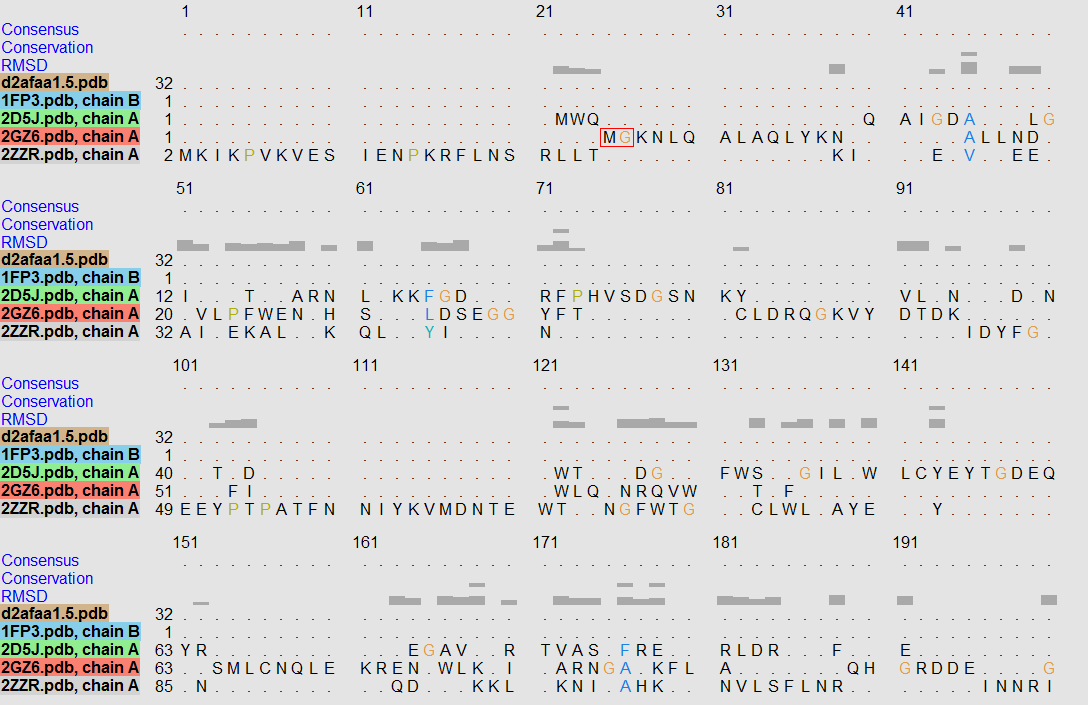

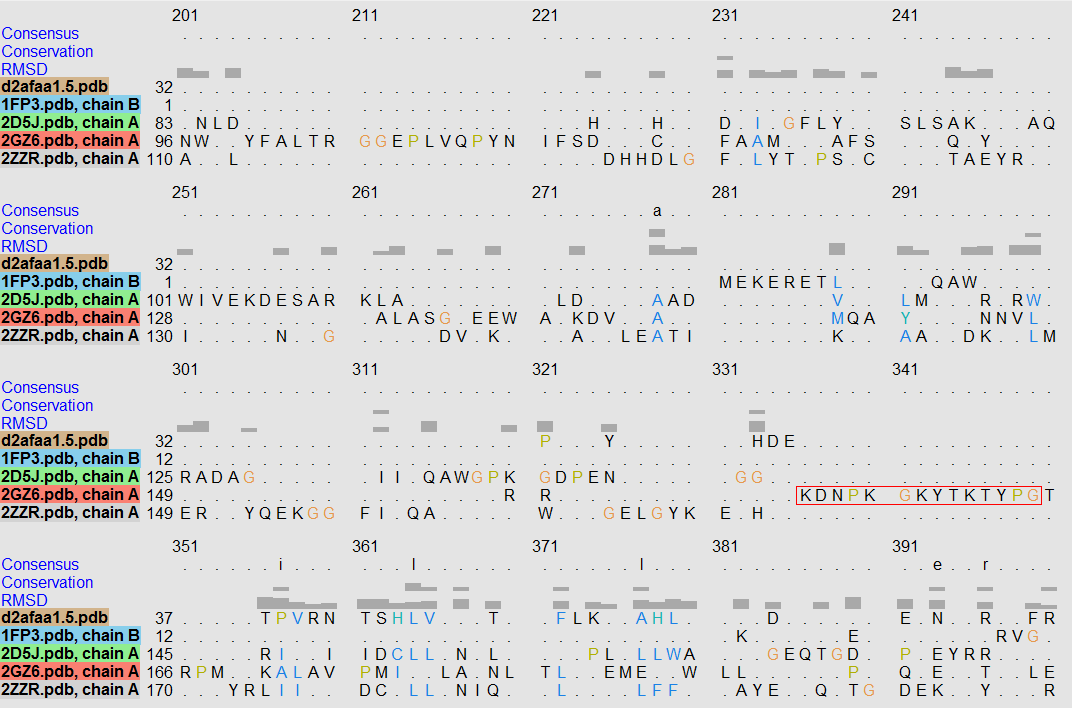
Fig S3

**
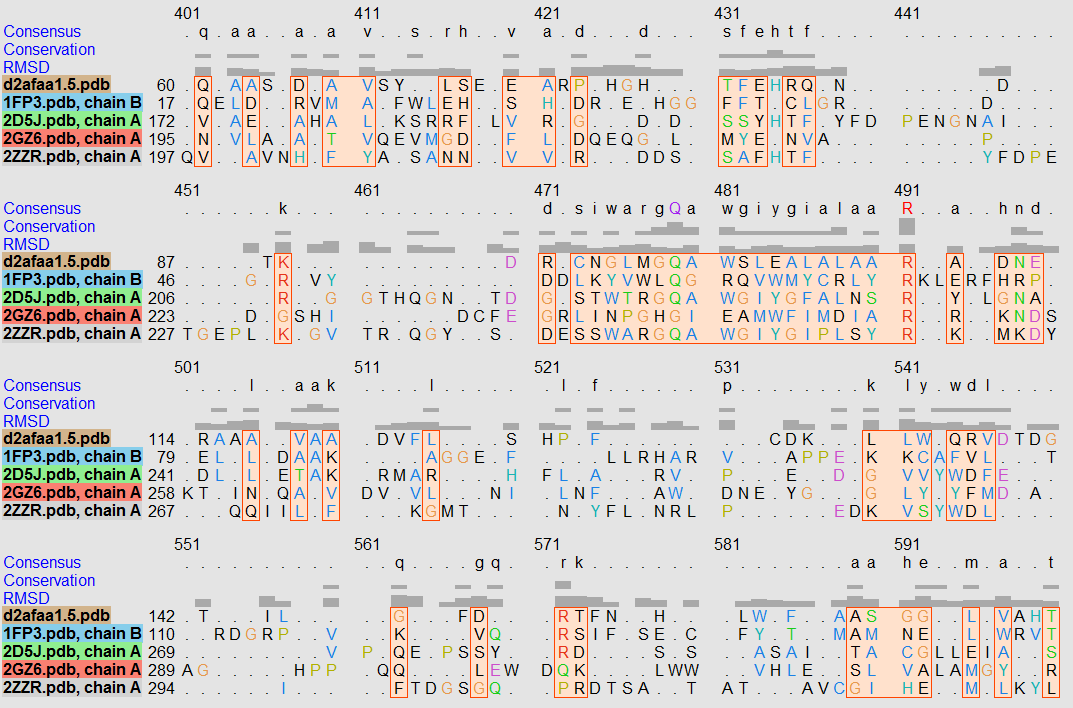

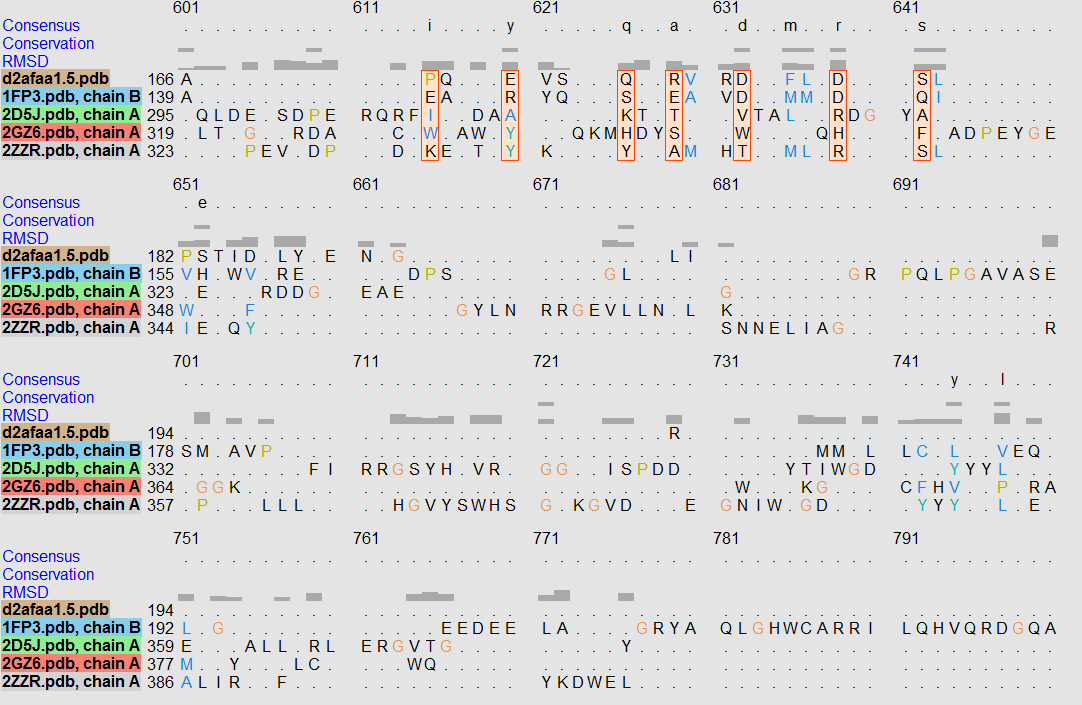
**

Supplement: Figure S3 — Alignment based on the structure of AglQ generated using pdb 2afa as template. A model of AglQ was created by the Chimera program using the structure of YihS (pdb 2afa) as template. A structure-based multiple alignment was generated using the Chimera program, with the positions of AglQ residues listed in the line listed as d2afaa1.5.pdb. The vertically framed and orange shaded residues represent overlapping positions in the first structure used to generate the alignment, while the horizontally framed residues represent those missing from the coordinates section of the pdb file. The residues are color-coded according to the Clustal X coloring scheme (www.jalview.org/help/html/colourSchemes/clustal.html). (DOC) [file pone.0081782.s003.doc]
