# Supplementary material for: AglQ Is a Novel Component of the Haloferax volcanii N-Glycosylation Pathway
Source: PLoS One. 2013 Nov 13;8(11):e81782. doi: 10.1371/journal.pone.0081782 (PMC3827465; doi:10.1371/journal.pone.0081782)
Supplement: Table S1 — Primers used for site-directed mutagenesis. (DOC) [file pone.0081782.s004.doc]

Table S1 – Primers used for site-directed mutagenesis

| **Name** | **Primer^1^** |
| --- | --- |
|  | |
| H34D | CACAACGGCCCGTAC**GAT**GATCCTGAAACACCTG |
|  | CAGGTGTTTCAGG**ATC**ATCGTACGGGCCGTTGTG |
| E37A | GGACACAACGGCCCGTACCATGAT**GCT**GAAACACCTGTTCGAAACACG |
|  | CGTGTTTCGAACAGGTGTTTC**AGC**ATCATGGTACGGGCCGTTGTGTCC |
| T38L | CAACGGCCCGTACCATGATCCTGAA**CTA**CCTGTTCGAAACACGT |
|  | ACGTGTTTCGAACAGG**TAG**TTCAGGATCATGGTACGGGCCGTTG |
| F50A | CTGAAGGCCCACGAACTTACG**GCT**GAAAATAGATTTAGACAAGCTGC |
|  | GCAGCTTGTCTAAATCTATTTTC**AGC**CGTAAGTTCGTGGGCCTTCAG |
| K52L | CACTGGTTAGTAACATTTCTG**AAG**GCCCACGAACTTACG |
|  | CGTAAGTTCGTGGGC**CTT**CAGAAATGTTACTAACCAGTG |
| E55K | GTAACATTTCTGAAGGCCCAC**AAA**CTTACGGATGAAAATAG |
|  | CTATTTTCATCCGTAAG**TTT**GTGGGCCTTCAGAAATGTTAC |
| D58A | GAAGGCCCACGAACTTACGGAT**GAA**AATAGATTTAGACAAGCTGC |
|  | GCAGCTTGTCTAAATCTATT**TTC**ATCCGTAAGTTCGTGGGCCTTC |
| E59A | CGTCTCACTGGTTAGTAACA**GCT** CTGAAGGCCCACGAACTTACG |
|  | CGTAAGTTCGTGGGCCTTCAG**AGC**TGTTACTAACCAGTGAGACG |
| R61D | CGAACTTACGGATGAAAAT**GAC**TTTAGACAAGCTGCTTC |
|  | GAAGCAGCTTGTCTAAA**GTC**ATTTTCATCCGTAAGTTCG |
| A66Q | GAAAATAGATTTAGACAAGCTCAGTCAGATGCGGTTTCTTATC |
|  | GATAAGAAACCGCATCTGA**CTG**AGCTTGTCTAAATCTATTTTC |
| H81D | GAGTGAAGAGGCACGACCA**GAT**GGCCATACGTTTGAACAC |
|  | GTGTTCAAACGTATGGCC**ATC**TGGTCGTGCCTCTTCACTC |
| K93A | CGTTTGAACACAGACAAAACGATACG**GCG**GACCGATGCAATGGCCTG |
|  | CAGGCCATTGCATCGGTC**CGC**CGTATCGTTTTGTCTGTGTTCAAACG |
| W104A | GCAATGGCCTGATG GGACAGGCA**GCG**TCACTTGAGGCCCTTGCGC |
|  | GCGCAAGGGCCTCAAGTGA**CGC**TGCCTGTCCCATCAGGCCATTGC |
| R114D | GTTGCGCTTGCAGCT**GAC**GCACTTGACAACGAACGTGCTGCTGC |
|  | GCAGCAGCACGTTCGTT**GTC**AAGTGCGTCAGCTGCAAGCGCAAC |
| N118A | GCAGCTAGAGCACTTGAC**GCC**GAACGTGCTGCTGC |
|  | GCAGCAGCACGTTC**GGC**GTCAAGTGCTCTAGCTGC |
| D187K | CGTGTTCGTGACTTTTTA**AAG**AGTCTTCCTTCAACTATCG |
|  | CGATAGTTGAAGGAAGACT**CTT**TAAAAAGTCACGAACACG |
|  | |

^1^ For each mutant, the forward and reverse primers used are listed. The mutant codon is indicated in bold.
